# Dual Electrophoresis Detection System for Rapid and Sensitive Immunoassays with Nanoparticle Signal Amplification

**DOI:** 10.1038/srep42562

**Published:** 2017-02-15

**Authors:** Fangfang Zhang, Junjie Ma, Junji Watanabe, Jinlong Tang, Huiyu Liu, Heyun Shen

**Affiliations:** 1 Beijing Key Laboratory of Bioprocess, Beijing Advanced Innovation Center for Soft Matter Science and Engineering, Beijing Laboratory of Biomedical Materials, College of Life Science and Technology, Beijing University of Chemical Technology, Beijing 100029, P. R. China; 2 Faculty of Science and Engineering, Konan University, 8-9-1 Okamoto, Higashinada, Kobe 658-8501, Japan

## Abstract

An electrophoretic technique was combined with an enzyme-linked immunosorbent assay (ELISA) system to achieve a rapid and sensitive immunoassay. A cellulose acetate filter modified with polyelectrolyte multilayer (PEM) was used as a solid substrate for three-dimensional antigen-antibody reactions. A dual electrophoresis process was used to induce directional migration and local condensation of antigens and antibodies at the solid substrate, avoiding the long diffusion times associated with antigen-antibody reactions in conventional ELISAs. The electrophoretic forces drove two steps in the ELISA process, namely the adsorption of antigen, and secondary antibody-labelled polystyrene nanoparticles (NP-Ab). The total time needed for dual electrophoresis-driven detection was just 4 min, nearly 2 h faster than a conventional ELISA system. Moreover, the rapid NP-Ab electrophoresis system simultaneously achieved amplification of the specific signal and a reduction in noise, leading to a more sensitive NP-Ab immunoassay with a limit of detection (LOD) of 130 fM, and wide range of detectable concentrations from 0.13 to 130 pM. These results suggest that the combination of dual electrophoresis detection and NP-Ab signal amplification has great potential for future immunoassay systems.

Immunosensors have been used extensively in a variety of fields such as clinical diagnosis, food security, environmental assessment, and drug assays. The signalling transfer strategies used in immunosensors are becoming more diverse, and include electrochemical-based methods, fluorescence immunoassays, surface plasmon resonance detection, silicon photonic micro-ring resonators, and other methods^1^. In the molecular diagnosis area, the fundamental goal is to achieve an optimum balance in three factors—fast, accurate, and cheap. However, it is currently possible to satisfy only two of these factors simultaneously^2^,^3^. The enzyme-linked immunosorbent assay (ELISA) is a common and reliable technology for protein detection and quantification that has been widely used in a variety of analytic fields^4^,^5^,^6^. However, the complete antigen-antibody reaction is achieved only after the diffusion of a sufficient number of antigen molecules toward the antibody-immobilized substrate; hence, long incubation times are the rate-limiting factor in the ELISA system, resulting in decreases in the assay sensitivity and the dynamic range^7^,^8^,^9^. Many research groups have dedicated much effort to the improvement of conventional ELISA systems. It has been shown that functional hydrophilic surface modification efficiently inhibits nonspecific protein adsorption^10^,^11^, the use of flow detection systems shortens detection times^12^,^13^,^14^, antibody-modified magnetic nanoparticles simplify the detection process^15^,^16^,^17^, and that secondary antibody- and enzyme-loaded nanoparticles enhance the detection sensitivity via signal amplification^17^,^18^,^19^.

Electrophoresis techniques can be used to separate individual proteins in a complex solution, and have been developed as semi-quantitative protein detection devices; such techniques include sodium dodecyl sulfate-polyacrylamide gel electrophoresis, and isoelectric focusing electrophoresis. Alternating-current or direct-current electrophoresis can easily be used to fabricate organic-inorganic hybrids, via ion deposition on a hydrogel template^20^,^21^,^22^. Cao and co-workers have developed electrophoresis titration devices for the rapid detection of protein content based on the principle of a moving reaction boundary, but the detection sensitivity is not sufficient for clinical diagnosis, unlike the conventional ELISA^23^,^24^.

In this study, we developed a rapid and sensitive dual electrophoresis immunoassay using a cationic polyelectrolyte multilayer (PEM)-modified filter (cellulose acetate, CA), leading to a dual flow detection system that overcame the diffusion reaction as the rate-limiting step (and hence avoided the major drawback of conventional ELISA systems). PEMs can be used to easily control the properties of arbitrary substrate surfaces; such properties include the hydrophobicity-hydrophilicity, charge properties, and morphology^25^. Hence, PEMs have been widely applied in the environmental, biomedical, and sensor fields^26^,^27^. Previously, we demonstrated that PEMs could easily control the protein adsorption properties, including the adsorption capacity and adsorption type from a mixed solution, via electrostatic interactions between the protein and the PEM surface^28^,^29^,^30^. Moreover, in traditional ELISA systems, positive PEM (poly(diallyldimethylammonium chloride) (PDDA)/poly(sodium 4-styrenesulfonate) (PSS))-modified polystyrene plates were used to block reagent enrichment (coverage, 100%) to efficiently inhibit nonspecific protein adsorption, improving the sensitivity of the conventional ELISA system^31^.

In this work, a PEM-modified filter adsorbed the primary antibody, and a blocking reagent was placed in a pair of glass cells; the sequential and directional migration of the antigen (Ag) and secondary antibody-immobilized polystyrene nanoparticle (NP-Ab) solutions toward the filter, driven by the electrophoretic force, induced rapid and sensitive Ag-Ab and NP-Ab-Ag reactions (Fig. 1). The dual electrophoresis detection system provided several advantages: (1) The primary antibody enrichment could be induced in the three-dimensional membrane filter, leading to a higher recognizing ability toward the antigen. (2) The two steps of the Ag-Ab reaction were carried out via the sequential electrophoresis of Ag and NP-Ab; hence, the Ag-Ab reaction time corresponded to the electrophoresis running time (2 min), and the total reaction time was nearly 2 h shorter than that of a conventional ELISA. (3) Because the NPs had a large specific surface area, the NP-Ab substantially increased the Ag-Ab reaction efficiency, and amplified the specific detection signal; further, the noise signal associated with NP-Ab was decreased by the short electrophoresis detection times. Combined, these factors provided a novel, rapid, sensitive flow immunoassay. The limit of detection (LOD) for mouse antibody was 130 fM, two orders of magnitude lower than that of a conventional ELISA. The results for this electrophoresis system suggested that complex serum samples could be simultaneously subjected to separation and detection processes, if the electrophoresis parameters were suitable for the differential rates and directional movement of mixed proteins, and the molecular cutoff of the filter was appropriate for antigen separation.

## Results and Discussions

### Protein Enrichment on the PEM-Modified Filter

A number of PEM studies have considered the higher sensitivity of immunosensors based on the control of protein adsorption using PEMs^32^,^33^. We previously investigated the detection sensitivity of one to ten layers of PDDA/PSS PEM in a conventional ELISA system^31^. The positive seven-step (PDDA/PSS)_3_PDDA proved to be the optimal PEM, in terms of both the uniformity of the PEM surface, and the detection sensitivity. A (PDDA/PSS)_3_PDDA PEM-modified porous membrane (LbL-CA) contributed toward the rapid and sensitive performance of a flow detection device using a centrifugal process^34^.

UV-vis absorbance experiments performed at 225 nm were used to monitor the PSS deposition, and the thickness of the seven-step PDDA/PSS PEM was approximately 7 nm (Fig. S1)^30^. Table 1 summarizes the results for the amount of primary antibody and blocking reagent (OVA) adsorption, and their coverage on the CA and LbL-CA filters, and the PS plate. Because of electrostatic interactions on the PEM surface, the amounts of primary antibody and OVA adsorbed on LbL-CA were approximately three times higher than the amounts adsorbed on the CA membrane. Moreover, the coverage was well over 100 of the values calculated from the position of the proteins on the apparent surface area, for both proteins; these results indicated that the proteins adsorbed within the membrane pores, that was investigated by gold colloid-labelled IgG adsorption within the pores of the membrane filter^34^. The amount of IgG adsorbed on LbL-CA was greater than the amount adsorbed on CA, which was notable in that the PEM completely modified the inner surface of the membrane pores. Therefore, the LbL-CA was able to contribute to a high-performance immunoassay, because of the high degree of enrichment of the primary antibody and OVA on/into the 3D membrane filter, which efficiently enhanced the specific signal (antigen molecular capture), and suppressed nonspecific signals (noise), respectively.

### Optimal NP-Ab for Electrophoresis Detection

Motivated by a consideration of flow detection, we selected the diameter of the PS NPs as 200 nm, and the pore size in the CA membrane filter as 450 nm. The Ab protein could be immobilized on the NPs via the carboxyl groups on the NP surface. First, we used B*γ*G to optimize the antibody concentration for covalent immobilization on the NPs. Figure 2(a) shows the amount of B*γ*G immobilization on the NPs with different protein and NP concentrations. When the protein concentration was increased, the standard deviation in the protein immobilization became large; the largest immobilized quantity was not obtained with the highest protein concentration (240 *μ*g/mL), because the protein easily self-linked at high concentrations. The highest immobilized quantity of protein was obtained using 60 *μ*g/mL B*γ*G and 1 mg/mL NP. Thereafter, these optimal conditions were used for the covalent immobilization of the secondary Ab on the NPs.

The concentration of secondary Ab is a very important factor for the sensitivity of the ELISA system, because of its direct effect on the specific signal and noise levels. Therefore, the concentration of NP-Ab was first optimized for different antigen concentration by considering the signal-to-noise ratio (S/N) (Fig. 2(b)). We selected three concentrations, 50, 100, 200 *μ*g/mL, of NP-Ab. When the concentration of NP-Ab was 100 *μ*g/mL, the S/N values were the highest over the full range of antigen concentrations. At concentrations lower than 100 *μ*g/mL, the antigen molecules could not be recognized sufficiently, resulting in decreases in the specific signal. On the other hand, at concentrations higher than 100 *μ*g/mL, nonspecific NP-Ab adsorption increased, resulting in increases in noise. Therefore, an optimal concentration of secondary antibodies offered the best balance of specific signal and noise, to obtain the highest sensitivity in the detection system. SEM imaging showed that the morphology of the PS NPs did not change after immobilization the Ab on the NPs (Fig. 2(c,d)). Before and after immobilization, the NPs were approximately 200 nm in diameter, as confirmed by dynamic light scattering measurements (Fig. S2).

### Conventional ELISA detection with NP-Ab signal amplification

First, we investigated the effect of NP-Ab signal amplification in a conventional ELISA system. Figure 3(a) shows results using a traditional ELISA method for mouse IgG detection on a 96-well PS plate. The standard deviations in the specific signal (absorbance) were very low over the full range of Ag concentration, and the correlation coefficient (*R*^2^) of the detection curve was excellent. However, the antigen concentration detection range was narrow, only three orders magnitude, and the LOD was 33 pM. When NP-Ab was used instead of secondary antibody, the antigen concentration detection range increased to five orders of magnitude, and the LOD was 0.33 pM, significantly lower than for the conventional ELISA system (Fig. 3(b)). However, the change in the specific signal with changes in the antigen concentration was not significant; that is, the slope of the detection calibration curve was not large. This result suggested that the steric hindrance effects of NP-Ab were greater than that of a single secondary Ab for the Ab-Ag reaction over sufficient incubation times, resulting in little signal change over the full range of antigen concentration.

### Dual electrophoresis ELISA detection with NP-Ab signal amplification

The number of captured Ag is linearly proportional to the Ab concentration, and increases linearly with time. To increase the sensitivity without extending the reaction time, one must apply either an amplification process or a condensing process for the Ab or for the Ag. In our previous work, we demonstrated that the electrophoretic force could drive the directional migration of Ag molecules, as well as local condensation, and the specific reaction with primary Ab, to achieve a rapid Ag-Ab reaction, providing a rapid and sensitive ELISA detection system^35^. Based on these results, we considered whether we could produce a two-step electrophoresis system for both the antigen and secondary antibody reactions, further improving the rapid immunoassay. Therefore, in this study, we investigated the dual electrophoresis of antigen and NP-Ab, to not only greatly shorten the reaction time, but also to significantly increase the specific signal via NP-Ab amplification.

**Figure 1:**
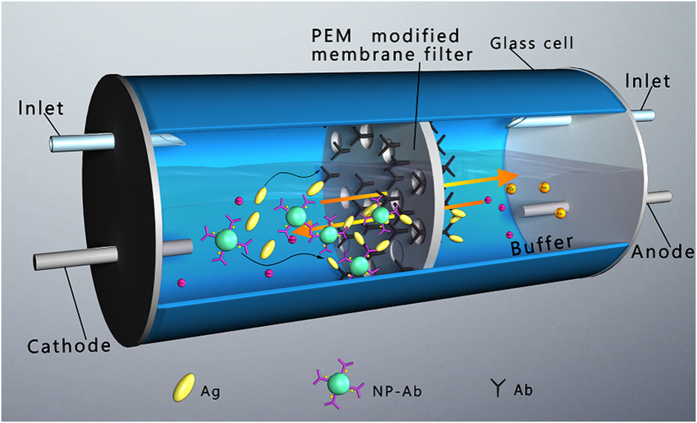
Illustration of rapid electrophoresis immunoassay. Antigen and antibody immobilized nanoparticles passively migrate to induce antigen-antibody reaction in 3D filter by an electrophoresis-driven protocol.

**Figure 2:**
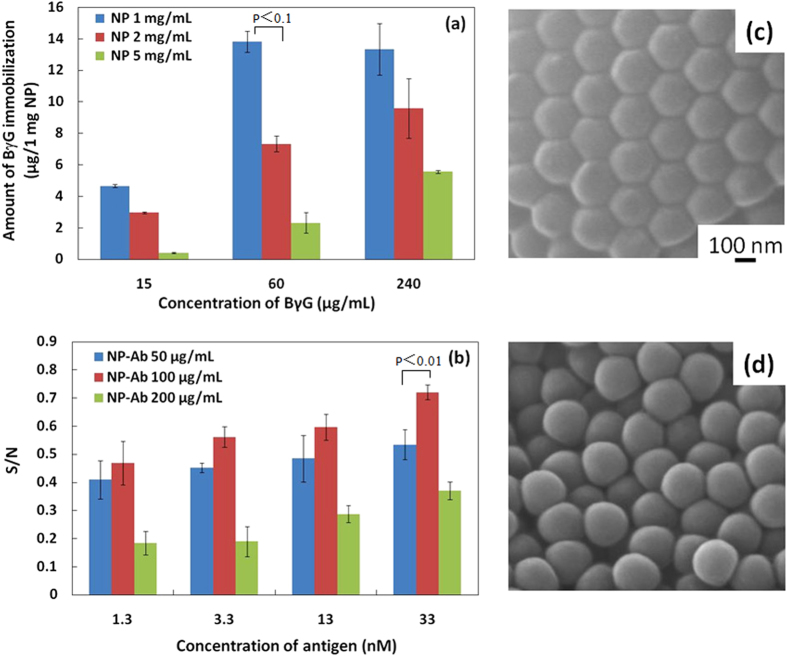
Characteristics of Ab immobilization on the PS NPs. (**a**) Amount of BγG immobilization on the NPs with different protein and NP concentrations (n = 3). (**b**) Optimal concentration of NP-Ab in the conventional ELISA system by the signal-to-noise ratio (S/N) (n = 3). Scanning electron microscope imaging of PS NPs (**c**) and NP-Ab (**d**). The scale bar is 100 nm. The covalent immobilized concentration of Ab and NPs were 60 *μ*g/mL and 1 mg/mL, respectively.

As shown in Fig. 1, the LbL-CA filter with adsorbed primary Ab and OVA (blocking reagent) was fixed between a pair of glass cells, and platinum electrodes were inserted on the two sides of the glass cells. An Ag solution (mouse IgG) and A phosphate buffer (PBS, pH 7.4) were injected separately into the cells, and a cathode and anode were inserted into the Ag solution and PBS cells, respectively, resulting in the directional migration of Ag molecules and NP-Ab toward the primary Ab adsorbed on the filter. Previously, we confirmed that the change in the specific signal on the LbL-CA filter was larger compared with the CA filter for the same range of antigen concentration; hence, we used the LbL-CA filter in all of the characterization performed in this study. We optimized the electrophoresis conditions for the NP-Ab-Ag reaction, taking account of the effects of running voltages and times on the S/N (Fig. 4). The different voltages and thus electrophoretic forces produced different velocities in the antigen molecules, in addition to inducing a frictional force between the antigen and the filter. The highest S/N value, and a relatively small standard deviation, was obtained using electrophoresis parameters of 30 V, 2 min. Because the Ag molecules could not efficiently react with the NP-Ab for detection times shorter than 1 min, a lower specific signal was observed for 1 min than for 2 min of detection. In the case of 5 min of detection, the noise was higher than the specific signal, resulting in a lower sensitivity than with 2 min of detection (Fig. S3).

**Figure 3:**
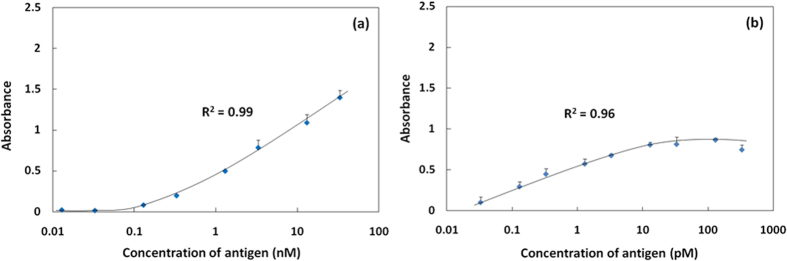
The specific signal with a change in the antigen. (**a**) The conventional static ELISA system on PS plate at 37 °C for 60 min (n = 3). (**b**) The conventional static ELISA system by NP-Ab detection on LbL-CA with 37 °C for 60 min (n = 3).

**Figure 4:**
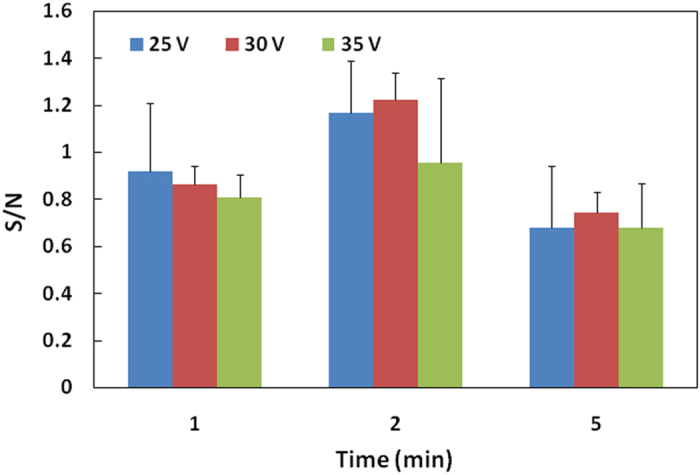
Optimal conditions of NP-Ab electrophoresis for mouse IgG detection by the specific signal-to-noise ratio (S/N) (n = 3).

**Figure 5:**
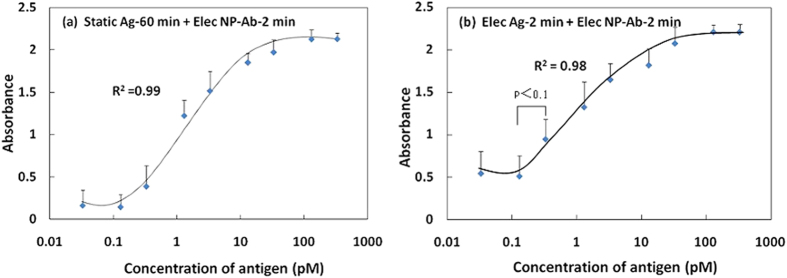
The specific signal with a change in the Ag by NP-Ab electrophoresis detection for 2 min. (**a**) Ag-Ab reaction is the static conventional method at 37 °C for 60 min; (**b**) Ag-Ab reaction is the electrophoresis method at r.t. for 2 min (n = 3).

Figure 5(a) shows the specific signals obtained using static Ag detection performed for 60 min, and NP-Ab electrophoresis detection performed for 2 min with changes in the Ag concentrations under optimal conditions. Compared with NP-Ab detection in the conventional ELISA system (Fig. 3(a)), a much higher specific signal change was obtained in the electrophoresis detection system; the change in absorbance was approximately twice that observed for the conventional ELISA system. Using growth/sigmoidal fitting, the *R*^2^ of the calibration curve was determined to be 0.99. The LOD was 130 fM, achieving the same level of NP-Ab detection as the conventional ELISA system, but the detection time was shorter by approximately 1 h. To further increase the detection rate, we fabricated the dual electrophoresis detection system, in which both Ag and NP-Ab were driven under electrophoresis, to induce directional migration and local condensation toward the membrane filter (Fig. 5(b)). Notably, we discovered that the dual electrophoresis detection system did not compromise the sensitivity; the two reaction steps took only 4 min, almost 2 h faster than a conventional ELISA system. The specific signal change and antigen concentration range were large, the same as for the one step electrophoresis detection system, with a LOD of 130 fM. The absorbance of LOD had significant difference from that of adjacent concentration point. This result indicated that the optimal electrophoretic force did not influence the stability of the Ab and Ag on the filter, but efficiently drove the directional migration of Ag and NP-Ab toward the primary Ab-immobilized filter to achieve a rapid and sensitive Ag-Ab reaction.

**Figure 6:**
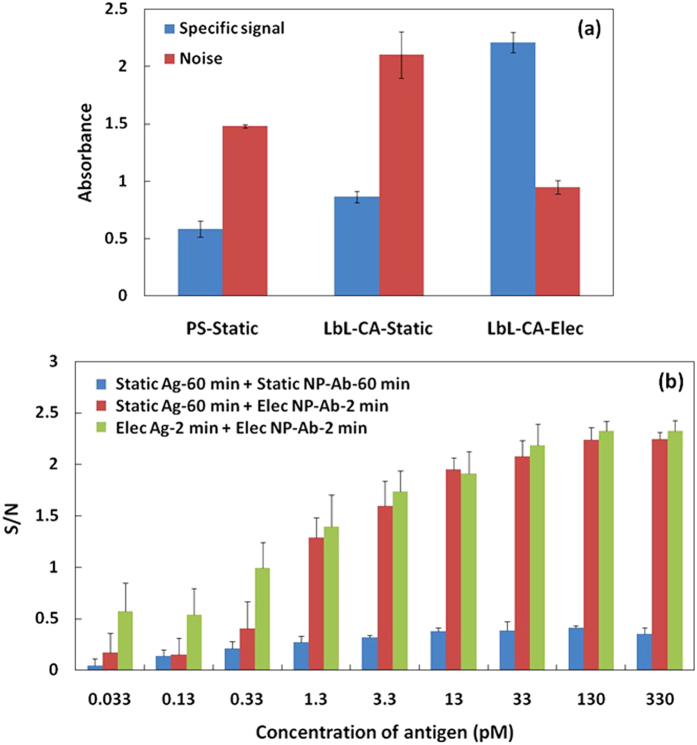
(**a**) The specific signal and noise by NP-Ab detection on different substrate with the conventional static (60 min) or dual electrophoresis (2 min) detection methods. The specific signal is in antigen concentration of 0.13 nM (n = 3). (**b**) The S/N of different concentrations of antigen for different detection methods on LbL-CA filter. ‘Static Ag-60 min + Static NP-Ab-60 min’: the conventional static ELISA method by NP-Ab detection for 60 min; ‘Static Ag-60 min + Elec NP-Ab-2 min’: Ag-Ab reaction is the static conventional method for 60 min and NP-Ab-Ag reaction is electrophoresis detection method for 2 min; ‘Elec Ag-2 min + Elec NP-Ab-2 min’: Ag-Ab and NP-Ab-Ag reaction both are the electrophoresis detection method for 2 min (n = 3).

To clarify the mechanism responsible for the high performance of the electrophoresis detection system, we compared the specific signal and noise of the NP-Ab detection with static and electrophoretic detection on a different substrate (Fig. 6(a)). ‘Static detection’ corresponded to conventional ELISA detection, with 60 min of incubation for the Ag-Ab reaction. ‘Electrophoretic detection’ was carried out using the dual electrophoresis detection system, with 2 min of incubation for the Ag-Ab reaction. The bar graph in Fig. 6(a) clearly shows that for ‘Static detection’, the noise approximately was two times higher than the specific signal; in contrast, the noise was half of the specific signal for ‘Electrophoretic detection’. Moreover, the specific signal and noise for ‘Electrophoresis detection’ were approximately three times and one-third of the values for ‘Static detection’ on the LbL-CA substrate, respectively. This result strongly demonstrated that although NP-Ab could significantly amplify the specific signal, the noise was increased with increasing incubation time. Therefore, the ‘Electrophoretic detection’ system not only provided sensitive detection in a short time, but also efficiently decreased the noise to induce a much higher specific signal.

We further investigated the detection sensitivities of different detection methods; S/N over the full range of Ag concentration are shown in Fig. 6(b). The S/N values for dual electrophoresis detection (‘Elec Ag-2 min + Elec NP-Ab-2 min’) were approximately four to six times higher than those obtained using the static conventional ELISA method (‘Static Ag-60 min + Static NP-Ab-60 min’). Notably, there was no significant difference (p > 0.1) between the S/N values for one-step electrophoresis (‘Static Ag-60 min + Elec NP-Ab-2 min’) and dual electrophoresis detection, for Ag concentrations from 1.3 to 330 pM. This result strongly demonstrated that the dual electrophoresis detection system was as stable and reliable as one-step electrophoresis detection over the full range of Ag concentrations; the detection time was nearly 1 h shorter for one-step electrophoresis detection, compared with the static method, because the diffusion limit associated with the static detection method was overcome. Moreover, the static conventional ELISA method showed high noise values for NP-Ab detection (Fig. 6(a)), while the dual electrophoresis detection system possessed much higher sensitivity (S/N) for each antigen concentration. Because in the dual electrophoresis process, directional migration of the NP-Ab, and local enrichment toward the Ag-immobilized LbL-CA filter, was induced by the electrophoretic force (in a similar fashion to Ag electrophoresis detection^35^), leading efficient reduction of the Ag-Ab reaction time. Furthermore, the rapid detection facilitated a decrease in nonspecific NP-Ab adsorption; hence, the dual electrophoresis detection system simultaneously achieved specific signal amplification and noise reduction, in contrast with the conventional ELISA system. We suggest that the combination of NP-Ab detection and electrophoretic techniques provides an optimal approach for facilitating high performance immunoassays.

## Conclusion

A rapid and sensitive dual electrophoresis immunoassay was developed based on an LbL-CA membrane filter. The application of an electrophoretic force resulted in the directional migration of Ag and NP-Ab, and their local condensation near the primary Ab-immobilized filter substrate, leading to rapid detection in 4 min, approximately 2 h shorter than a conventional ELISA system. Moreover, the rapid NP-Ab electrophoresis detection not only amplified the specific signal, but also decreased the noise signal, as a result of the highly rapid electrophoresis detection, which induced sensitive NP-Ab signal amplification detection, with an LOD of 130 fM. This combined system comprising dual electrophoresis detection and NP-Ab signal amplification can contribute to novel, rapid, and sensitive immunoassay fabrication.

## Methods

### Materials

PDDA (#17338; Polyscience Inc., Niles, IL, USA; Mw = 2.4 × 10^5^ g/mol) and PSS (#416888; Alfa Aesar, Ward Hill, MA, USA; Mw = 7.0 × 10^4^ g/mol) were used as polyelectrolytes. The diameter and pore size of the CA membrane filter (C020A045A; Advantec Toyo Roshi Kaisha, Ltd., Tokyo, Japan) were 13 mm and 450 nm, respectively. Rabbit anti-mouse IgG (M7023), mouse IgG (I5381), goat anti-mouse IgG-horseradish peroxidase conjugate (goat antimouse IgG-HRP; A4416), ovalbumin from chicken egg white (OVA, A5503) and N-Hydroxysuccinimid (NHS) were acquired from Sigma, St Louis, MO, USA. 1-ethyl-3-(3-dimethylaminopropyl) carbodiimide hydrochloride (EDC) was purchased from Dojindo Co. Ltd., Shanghai, China. Disodium hydrogen phosphate and monometallic sodium orthophosphate were purchased from Beijing Chemical Reagent Co. Ltd., Beijing, China. Carboxylated PS NPs (PS-01-200, diameter 200 nm) were purchased from Janus New-Materials Co. Ltd., Nanjing, China. Tris(hydroxymethyl) aminomethane hydrochloride (Tris-HCl; Acros Organics, Fair Lawn, NJ, USA) was used in the preparation of a buffer solution. The chemicals were used in the experiments without further purification. Ultrapure water was used throughout the experiments.

### Fabrication of PEM on the CA filter

The concentrations of the PDDA and PSS solutions were adjusted to 0.2 mg/mL by adding 50 mmol/L of Tris-HCl containing 0.15 mol/L of NaCl. Initially, the CA filter was immersed in the PDDA solution for 2 min at room temperature; subsequently, the filter was rinsed with ultrapure water for 30 s. Next, the filter was immersed in a PSS solution as described above. This alternating adsorption process was repeated for 3.5 cycles for the preparation of a positive seven-step (PDDA/PSS)_3_PDDA PEM on the filter.

### Antibody immobilization on PS NPs

The parameters of the PS NPs are shown in Supporting Information Table S1. 10 *μ*L, 10 mg/mL of EDC and NHS solution (phosphate buffer saline (PBS; pH 6.3)) were added to 80 *μ*L, 1 mg/mL PS NP solution (PBS, pH 6.3), respectively, and the resulting solutions were then incubated at room temperature for 20 min. Thereafter, the PS NPs were rinsed twice with PBS at pH 7.4, by applying centrifugation at 14000 rpm/14462 × g for 13 min. Bovine gamma globulin (B*γ*G) was used as a model protein to optimize the covalent immobilized condition of the secondary antibodies (anti-mouse IgG-HRP). 100 *μ*L of B*γ*G at concentrations of 15, 60, and 240 *μ*g/mL was added to different concentrations of PS NP (1, 2, and 5 mg/mL, respectively), and incubated at room temperature for 2 h. 100 *μ*L of a 5-mg/mL solution of a blocking reagent (OVA) was added to the NPs over a period of 1 h, while the NPs were incubated at room temperature, to block the unreacted NHS groups. Finally, the NPs were rinsed twice with PBS at pH 7.4, under centrifugation applied at 14000 rpm for 10 min. The optimized concentrations of secondary antibodies and NPs were 60 *μ*g/mL and 1 mg/mL, respectively. The OVA concentration was an important factor influencing the NP-Ab monodispersity. We investigated the effect of OVA concentration on the size distribution of NP-Ab (Supporting Information Table S2, Fig. S4). The change in size of the NPs before and after the antibody immobilization and Ag-Ab reactions on the NPs was also investigated (Supporting Information Table S3, Fig. S2). The diameter of the NPs clearly increased by approximately 20 to 30 nm after each step of protein immobilization, which indirectly demonstrated that the protein immobilization was successfully completed.

### ELISA Protocol for the Electrophoresis Detection System

The primary antibody (anti-mouse IgG, 60 *μ*g/mL) and OVA (1 mg/mL) were adsorbed onto the filter at 4 °C overnight, and at 37 °C for 1 h, respectively. Thereafter, the filter was rinsed, and the adsorbed protein was then removed via rinsing with 1 wt% of n-sodium dodecyl sulfate (Wako Pure Chemical Industries, Ltd., Osaka, Japan). The recovered protein was evaluated using a Micro BCA kit (Pierce, No. 23235, Rockford, IL, USA) at 570 nm, using a multi-well plate reader (PT-3502G, Beijing Potenov Technology Co. Ltd., Beijing, China).

For the electrophoresis system, the primary antibody and blocking reagent (OVA)-treated filter was placed between a pair of glass cells (Fig. 1). Platinum electrodes were inserted on the two sides of the glass cells, fixed by a rubber plug. PBS (1 mL, 0.03 M, pH 7.4) and antigen solution (mouse IgG; 1 mL) at 0.03 M PBS were separately injected into the cells. The inlet syringe needles were remained on the rubber plug for opening to atmosphere. The cathode and anode were connected to the antigen solution and PBS solution cells, respectively, resulting in directional migration of the antigen molecules toward the primary antibody adsorbed on the membrane filter. Finally, the specific signal was detected using 100 *μ*g/mL of NP-Ab (an optimized concentration) at 37 °C for 1 h, and was visualized using a reaction between tetramethylbenzidine (34028, 1-step ultra TMB-ELISA, Thermo Fisher Scientific Co. Ltd., Rockford, IL, USA) and HRP. A brief protocol for the conventional ELISA process is described in the Supporting Information.

## Additional Information

**How to cite this article**: Zhang, F. *et al*. Dual Electrophoresis Detection System for Rapid and Sensitive Immunoassays with Nanoparticle Signal Amplification. *Sci. Rep.* **7**, 42562; doi: 10.1038/srep42562 (2017).

**Publisher's note:** Springer Nature remains neutral with regard to jurisdictional claims in published maps and institutional affiliations.

## Supplementary Material

Supplementary Information

## Figures and Tables

**Table 1:** Results of the Primary Ab and OVA Adsorption.

Surface	Primary IgG^a^ (*μ*g/cm^2^)	IgG coverage^b^/(%)		OVA (*μ*g/cm^2^)	OVA coverage^c^/(%)	
		End-on	Side-on		End-on	Side-on
CA	0.42 ± 0.04	23	156	1.87 ± 0.40	445	693
LbL-CA	1.35 ± 0.04	73	500	5.54 ± 0.12	1319	2052

^a^The dimension was calculated from the apparent area of filter (diameter was 13 mm) (n = 3).

^b^The dimensions of the IgG are 14.5 × 8.5 × 4 nm^36^. When one assumes full monolayer coverage, the amount of IgG adsorption was 1.85 and 0.27 *μ*g/cm^2^ in the end-on and side-on positions, respectively.

^c^The dimensions of OVA are 7 × 5 × 4.5 nm^37^. When one assumes full monolayer coverage, the amount of OVA adsorption was 0.42 and 0.27 *μ*g/cm^2^ in the end-on and side-on positions, respectively.
