# Supplementary material for: Dual Electrophoresis Detection System for Rapid and Sensitive Immunoassays with Nanoparticle Signal Amplification
Source: Sci Rep. 2017 Feb 15;7:42562. doi: 10.1038/srep42562 (PMC5309740; doi:10.1038/srep42562)
Supplement: Supplementary Information [file srep42562-s1.doc]

**Supporting information**

**Dual Electrophoresis Detection System for Rapid and Sensitive Immunoassays with Nanoparticle Signal Amplification.**

**Fangfang Zhang^1^**^#^**, Junjie Ma****^1^**^#^**, Junji Watanabe^2^, Jinlong Tang^1^, Huiyu Liu^1^*, and Heyun Shen^1^***

^1^ Beijing Key Laboratory of Bioprocess, Beijing Advanced Innovation Center for Soft Matter Science and Engineering, Beijing Laboratory of Biomedical Materials, College of Life Science and Technology, Beijing University of Chemical Technology, Beijing 100029, P.R. China

^2^ Faculty of Science and Engineering, Konan University, 8-9-1 Okamoto, Higashinada, Kobe 658-8501, Japan

**Contents**

**Figure S1** UV-vis spectra obtained by stepwise assembly of PDDA/PSS multilayers

**Figure S2** The size distribution of PS NPs after Ab and Ag immobilization

**Figure S3** The speciﬁc signal and noise by NP-Ab electrophoresis detection in different times

**Table S1** The parameters of PS NP

**Table S2** The size of NP-Ab under different concentrations of OVA blocking

**Figure S4** The size distribution of NP-Ab with different concentration of OVA blocking

**Table S3** The size and zeta potential of NP after Ab and Ag immobilization

1. **UV-vis spectra obtained by stepwise assembly of PDDA/PSS multilayers**

The resulting LbL assemblies were identified from the change in absorbance as a function of the number of PSS layers (**Figure S-1**). To estimate total PSS deposition, the quartz plate was positioned vertically in a quartz cuvette containing 50 mmol/L of Tris-HCl (pH 7.4), and its UV-vis absorbance at 225 nm (light path = 10 mm) was measured. From this measured absorbance, the amount of PSS deposition during the LbL assembly was estimated using a standard absorbance curve of PSS solutions. Ishihara et al. reported that the standard curves of protein solutions were used to determine the amount of proteins adsorption on a substrate. [1] In their report, the amount of protein molecules adsorbed on the quartz plate was equivalent to that in the protein solution in the total light path.

**Figure S1**. UV-vis spectra obtained by stepwise assembly of PDDA/PSS multilayers on a quartz plate. Blue lines: PDDA layers; red lines: PSS layers.

1. **The size distribution of PS NP after Ab and Ag immobilization**

**Figure S2**. The size distribution of PS NPs after Ab and Ag immobilization.

1. **The speciﬁc signal and noise by NP-Ab electrophoresis detection in different times**

**Figure S3.** The speciﬁc signal and noise by NP-Ab electrophoresis detection in different times with 30 V voltage.

1. **The parameters of polystyrene nanoparticles.**

1. **The size of NP-Ab under different concentrations of OVA blocking.**

1. **The size distribution of NP-Ab with different concentration of OVA blocking.**

**Figure S4.** The size distribution of NP after Ab immobilization with different concentration of OVA blocking. Ab and NP concentrations are 60 μg/mL and 100 μg/mL, respectively.

1. **The size and zeta potential of NP after Ab and Ag immobilization.**

1. **Conventional ELISA Protocol (Sandwich Assay)**

The primary antibody (100 μL/well) was adsorbed onto 96 wells microplate at 4°C overnight to facilitate antigen detection. Next, bovine serum albumin (BSA) or OVA (1 mg/mL; 200 μL/well) as blocking reagent was adsorbed onto each substrate at 37°C for 1 h for inhibiting nonspecific protein adsorption (antigen or secondary antibody). Subsequently, antigen-primary antibody, secondary antibody-antigen reaction were carried out at 37°C for 1 h (100 μL/well), respectively. Finally, the enzyme linked on secondary antibody react with that of substrate induced color change to antigen quantification. The absorbance of the solution is measured by the multi-well plate reader. The microplate should be sufficiently rinsed after protein adsorption in every step.

**References**

[1] Ishihara, K.; Nomura, H.; Mihara, T.; Kurita, K.; Iwasaki, Y.; Nakabayashi, N. *J. Biomed. Mater. Res.* **1998,** *39*, 323.
